# The short-term effectiveness of antidepressants in a transdiagnostic sample and determinants of side effects and medication nonadherence: an observational study

**DOI:** 10.3389/fpsyt.2025.1752173

**Published:** 2026-01-12

**Authors:** Arda Bağcaz, Beren Özel

**Affiliations:** 1 Department of Psychiatry, Faculty of Medicine, Hacettepe University, Ankara, Türkiye; 2 Department of Psychiatry, Faculty of Medicine, Başkent University, Ankara, Türkiye; 3 Institute of Psychiatry, Psychology and Neuroscience (IoPPN), King’s College London, London, United Kingdom; 4 Department of Psychiatry, Mus State Hospital, Mus, Türkiye

**Keywords:** adverse effects, antidepressants, effectiveness, medication adherence, psychoeducation, real-world evidence, treatment discontinuation

## Abstract

Antidepressants are widely prescribed, yet evidence on their short-term effectiveness and tolerability in routine outpatient psychiatric care remains limited. This observational study followed 123 patients newly initiated or switched to an antidepressant in a university hospital outpatient clinic to examine early-term side effects, their associations with treatment discontinuation, and predictors of short-term response in a pragmatic routine-care setting. Depression and anxiety symptoms, sociodemographic variables, and functionality were assessed at baseline; side effects and adherence were evaluated through structured interviews during the first month; and treatment response was determined at follow-up. The remission rate at one month was 72.3%, while 12.3% showed no response. Higher pretreatment anxiety levels predicted greater severity of early side effects, and lower baseline functionality predicted poorer adherence. Patients who achieved response or remission were more likely to report at least one side effect. These findings suggest that many patients struggle to maintain antidepressant treatment during the early phase, and that anxiety and functional impairment influence both tolerability and adherence. Early psychoeducation, close monitoring, and tailored support for patients with elevated anxiety or reduced functionality may improve continuity of treatment and reduce the risk of delayed recovery.

## 1 Introduction

The consumption of antidepressants has increased by nearly 50% in Organisation for Economic Cooperation and Development (OECD) countries between 2011 and 2021 (1).

There is an increasing global trend in prescribing antidepressants, and most users have depressive disorders and anxiety disorders (2). In addition, the economic burden of antidepressant use has been rising since the COVID-19 pandemic (3). Therefore, treatment effectiveness has become increasingly important, as ineffective treatment attempts due to early discontinuation have led to delayed recovery and an increased economic burden due to either patients’ prolonged loss of functionality or higher pharmaceutical expenditures.

Recently, antidepressant drugs with diverse mechanisms of action have been in use (4, 5). Selective serotonin reuptake inhibitors (SSRIs) are the first-choice antidepressants due to their broad indications, followed by selective serotonin/noradrenaline reuptake inhibitors (SNRIs) as the second-most preferred option (6, 7). Although substantial evidence supports the efficacy of these drugs, their effectiveness in daily practice may be hindered by factors such as medication nonadherence, often driven by hesitations about long-term effects, intolerance to side effects, and additional sociodemographic and cultural factors (8–10).

While antidepressants are generally well-tolerated, gastrointestinal adverse effects, such as nausea, diarrhea, and constipation, as well as other side effects, including anxiety, insomnia, headache, and sexual dysfunction may occur, albeit within a short time (11). Frequently, the feeling of anxiety itself in patients with anxiety disorders, or the accompanying high levels of anxiety in other psychiatric disorders, can lead to treatment discontinuation (12). Moreover, the expected benefits of antidepressant treatment usually become evident only after several weeks (4). This combination of delayed response onset and the emergence of side effects often disrupt medication adherence (11). Although these adverse events may not always be severe, they may significantly influence a patient’s decision to discontinue medication. Reported discontinuation rates for antidepressants within the first three months range from 40% to 60% (13). Unless the clinical variables that hinder medication, adherence are understood, no matter how efficient an antidepressant is under ideal conditions, it will not ensure that it is effective in clinical use nor predict clinical success. Although studies on the efficacy and mechanisms of action of antidepressants are abundant (14) research on real-world experiences and effectiveness in daily psychiatric practice remains limited.

This study is a naturalistic follow-up of antidepressant treatments administered in a university hospital psychiatry outpatient clinic with a focus on early-term side effects and their impact on treatment discontinuation. We monitored the early-term side effects in patients initiating antidepressant treatment for the first time or undergoing changes in their regimen. We aimed to determine the relationship between the presence, type, and severity of the side effects and treatment discontinuation. At the same time, we examined the associated factors of side effect severity, including age, sex, diagnosis, the severity of initial anxiety and depression symptoms, comorbidities, and the medications used.

## 2 Materials and methods

Patients aged 18 years or older who applied to the university hospital psychiatry outpatient clinic and were initiated on a new antidepressant medication or whose antidepressant regimen was recently recommended to be changed by the evaluating psychiatrist were enrolled in the study. Refusal to consent and the presence of intellectual disability, dementia, or psychotic disorders were determined as exclusion criteria. At the start of the follow-up, 132 patients were evaluated, and 123 meeting inclusion criteria were included in the study. One hundred patients attended their scheduled phone interviews for the side effect screening, and 65 patients came for their first-month check-ups and completed the study follow-up.

Each patient was interviewed for 20 minutes, and their sociodemographic information, past and current psychiatric disorders, comorbid physical diseases, the diagnosis based on an unstructured clinical interview by the psychiatrist that led to the initiation/change of antidepressants (according to ICD-10: panic disorder, generalized anxiety disorder, social phobia, major depressive disorder, obsessive-compulsive disorder, post-traumatic stress disorder, somatization disorder, and conversion disorder), current medications, the newly prescribed antidepressant, and its dosage were recorded. Functionality was assessed by asking about different areas of their lives, including work, family, and social relationships, using a 5-point Likert-type numerical scale (0 = no impairment, 4 = severe impairment). At the end of the interview, patients completed the Beck Depression Inventory-II (BDI-II) and the Beck Anxiety Inventory (BAI) (15, 16).

Patients were then scheduled for a 10-minute phone interview between the 14th and 20th days after starting treatment. Patients were asked about treatment adherence in this interview, and side effects were assessed using the Udvalg for Kliniske Undersogelser Side Effect Rating Scale (UKU). The severity and whether they interfered with treatment continuation were noted for any reported side effects. Additionally, patients were asked about their Patient Information Leaflet (PIL) -reading habits and whether they reached or needed to reach their doctor during this period.

**Figure 1:**
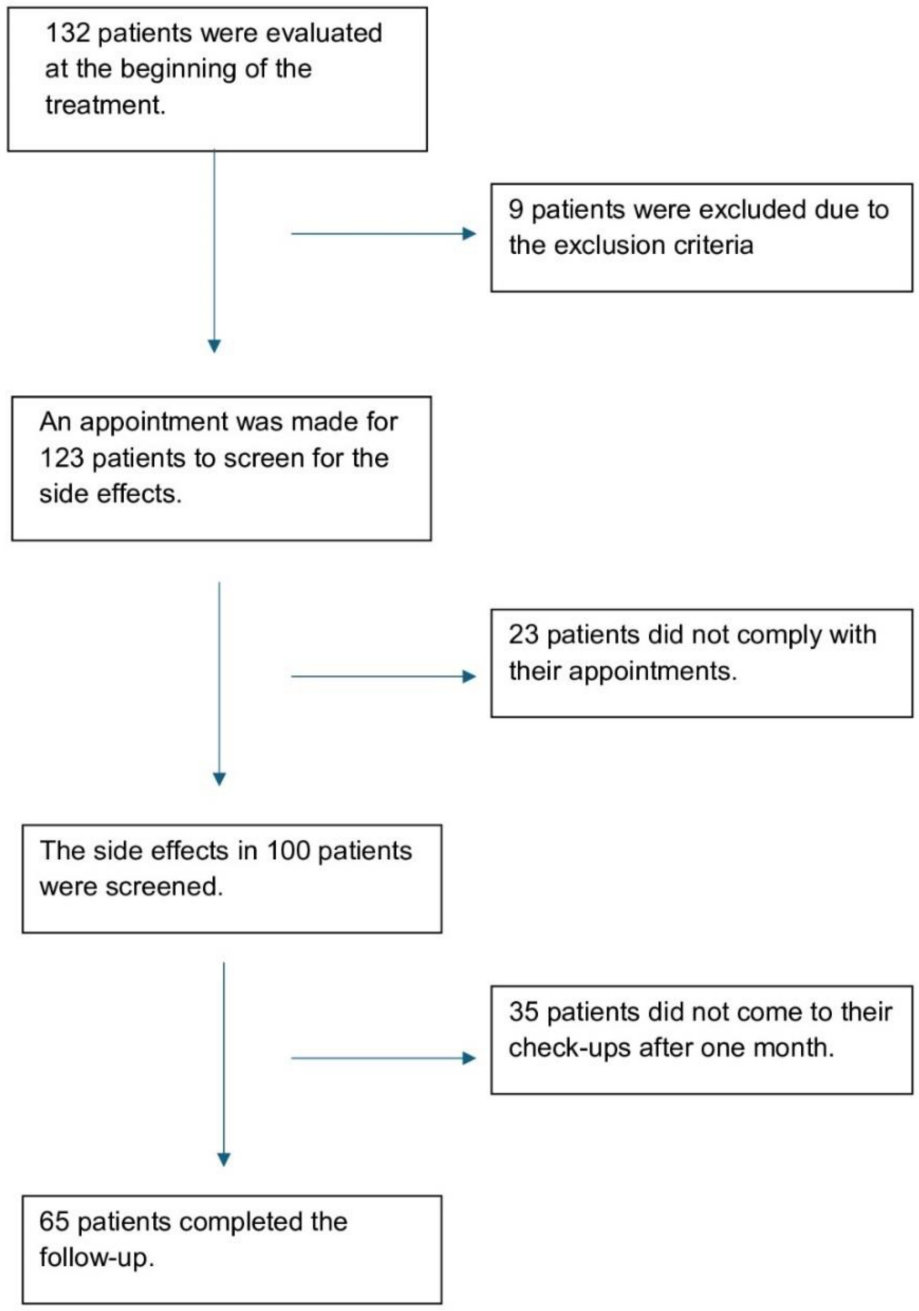
The flowchart of the study.

Patients were informed about the study design in advance. As this was an observational-naturalistic study, no interventions or treatment changes were made. If participants requested or required such changes, they were referred to the evaluating psychiatrists. The flowchart is provided in **Figure 1**.

At the follow-up interviews done between the 4th and 6th weeks of the treatment, symptom severity and functionality were reassessed, and early-term treatment responses for the patients with depressive disorders and anxiety disorders were determined. We defined response as a reduction of a minimum of 25% in BDI-II for depressive symptoms and BAI scores for anxiety symptoms, indicating at least minimal clinical improvement. This threshold aligned with previous studies, including drug trials (17), whereas some other studies adopted higher cutoff points (e.g., 50% improvement) (18, 19). Since these higher cutoff points were usually used to determine a broader concept of “difficult-to-treat depression” (20), we opted to use the 25% threshold to focus on early, short-term treatment responses in our sample. We defined remission as a BDI-II score <9 for depression and a BAI score <8 for anxiety disorders at the second evaluation (21).

Finally, self-report scales were repeated. We used the total score of the Beck Anxiety Inventory, which was developed by Beck et al. (15) and adapted to Turkish by Ulusoy et al. (22), to determine anxiety symptom levels (22). It consists of 21 questions, scored between 0 and 3. Total score ranges from 0 to 63. We used the Beck Depression Inventory-II to determine the depressive symptom levels. Kapci et al. (23) showed the validity and reliability of its Turkish version (23). Finally, the UKU Side Effect Rating Scale was used to detect the presence and severity of side effects. This is an instrument developed to scan the side effects of psychotropic agents used (24). It comprises 48 items evaluating psychological, neurological, autonomic, and other side effects, and the severity is rated between 0 and 3. In our study, the scores are grouped based on 0 scores for none, 1 for mild, 2 for moderate, and 3 for severe.

The data were analyzed using IBM SPSS Statistics Version 23.0. The relationships between the severity of side effects, discontinuation of treatment and sociodemographic variables, anxiety, depression, and functionality scores at first admission, comorbid physical diseases, drug classes used, and scale scores in the follow-up were analyzed. Groups with and without response to treatment, diagnostic groups (major depressive disorder, anxiety disorders (panic disorders, generalized anxiety disorder, and social anxiety disorder) and others), and groups that continued and ceased the treatment were compared in the presence of side effects, the severity of side effects, accompanying depression levels, accompanying anxiety levels, functionality levels, sociodemographic characteristics, and drug classes used. Categorical variables were compared using the chi-square test, and continuous variables were compared with the Student’s T-test or Mann -Whitney U for independent groups by checking whether they were normally distributed. Effect sizes were calculated for key categorical outcomes using Cramer’s V. In addition, the relationships between the severity of side effects and other variables were examined with Pearson correlation analyses. A linear regression analysis was conducted to investigate the determinants of side effect severity. Variables included in the analysis were determined based on study objectives and existing literature. Skewness and kurtosis tests were performed to test the model’s suitability for linear regression analysis and the normality of the residuals. The Durbin-Watson test was performed to control for autocorrelation. Multicollinearity severity was tested by variance inflation factors (VIF) and tolerance of variables. Finally, a logistic regression analysis was done to examine the determinants of discontinuation. A result of p<0.05 was accepted to indicate statistical significance.

## 3 Results

### 3.1 The sociodemographic and clinical characteristics of sample

**Table 1** presents the characteristics of the sample. The mean age of the sample was 35.5 (SD = 15.93) and no differences were detected between diagnostic categories in age (t = 0.748, df = 111; p = .456). The dropout rate was 18.7% before side-effects screening and 47.2% for the entire follow-up. The participants who completed the follow-up or dropped out were compared in sex, age, diagnoses, antidepressant categories, comorbidities, additional drug use, and treatment status. The dropped-out patients were older (t = 2.681, df = 110.856; p = .008), their depression and anxiety symptom severities were lower (respectively, t = -3.430, df = 115.738; p = .001; t = -2.705, df = 120; p = .008), and the additional benzodiazepine use was lower (×2 = 8.591, df = 1; p = .003) than the patients who completed the follow-up.

**Table 1:** The features of the sample and treatment.

Variables	Anxiety disorders (n = 49) n (%)	Major depressive disorder (n = 64) n (%)	Other diagnoses (n=10) n (%)	Total	×2, df; p
Sociodemographic and Clinical Features (N = 123)					
Sex (Women)	34 (69.4)	46 (71.9)	6 (60.0)	86 (69.9)	0.591, 2;.744
Physical Comorbidity (Yes)	11 (22.4)	22 (34.4)	1 (10.0)	34 (27.6)	3.667, 2;.160
The features of the treatment (N = 123)					
Antidepressant Class (SSRI)	44 (89.8)	47 (73.4)	10 (100)	101 (82.1)	7.427, 2;.024*
Treatment Status (Beginning New Antidepressant)	42 (85.7)	50 (78.1)	8 (80.0)	100 (81.3)	1.064, 2;.588
Psychotropic Combination (Yes)	15 (30.6)	24 (37.5)	3 (30.0)	42 (34.1)	0.669, 2;.716
Additional Nonpsychotropic Drugs (Yes)	11 (22.34)	20 (31.3)	1 (10.0)	32 (26.0)	2.567, 2;.277
Other variables about the treatment process (N = 100)					
Did the patient read the prospectus? (Yes)	10 (26.3)	26 (47.3)	3 (42.9)	39 (39.0)	4.196, 2;.123
Did the patient need to reach his/her doctor? (Yes)	10 (23.7)	21 (38.2)	2 (28.6)	33 (33.0)	1.498, 2;.473
Was the patient able to continue the treatment as recommended? (Yes)	35 (91.9)	43 (78.2)	7 (100)	85 (85.0)	4.745, 2;.093

*p<.05.

### 3.2 Antidepressant preferences

**Figure 2:**
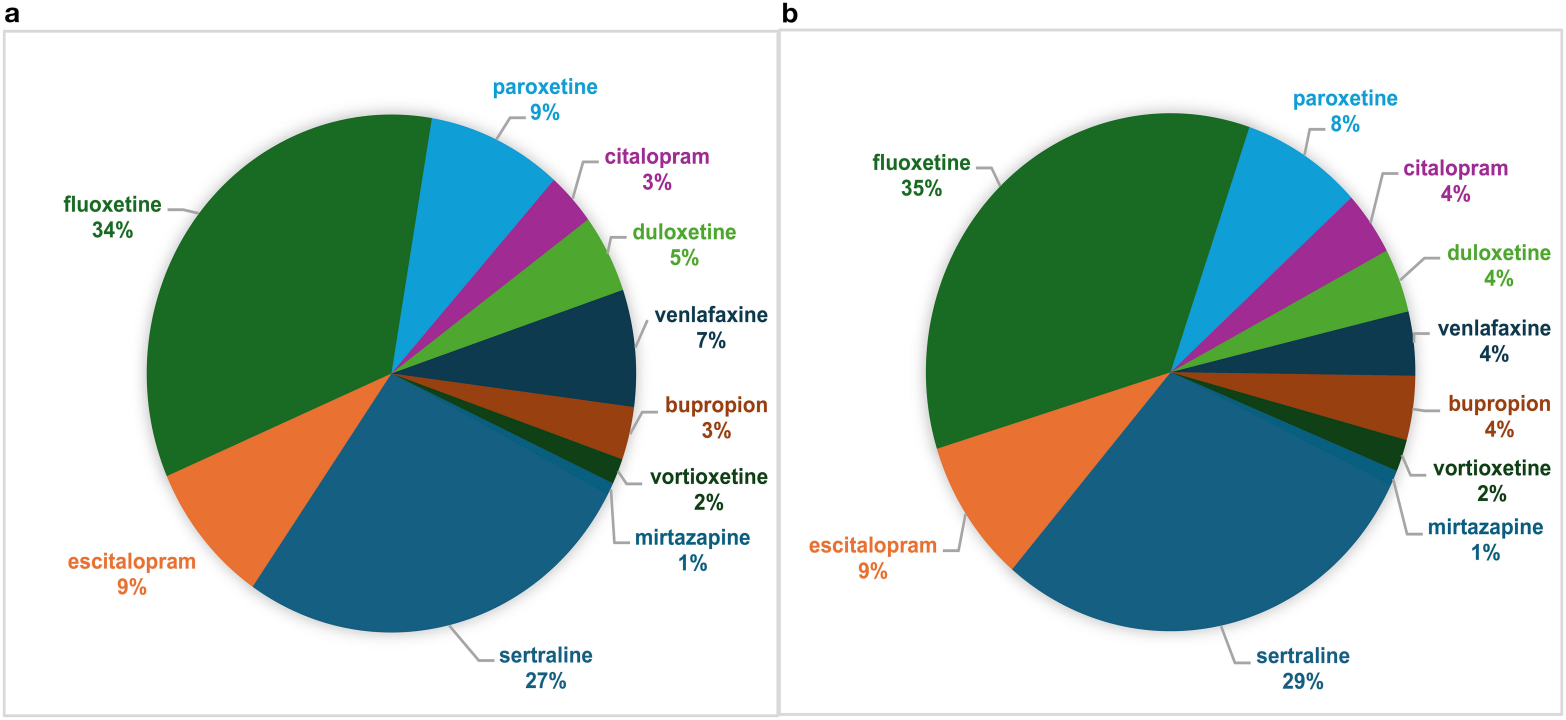
**(a)** Distribution of antidepressants prescribed for the whole group. **(b)** Distribution of antidepressants among patients initiating an antidepressant therapy first.

The recommended antidepressants are shown in **Figure 2**. Fluoxetine was the most prescribed antidepressant for depression patients (40.6%), for the whole group (34.1%), and for the patients starting a new treatment (35.7%). Sertraline was the most preferred antidepressant for anxiety disorders (38.8%). Drug classes other than SSRIs were found to be prescribed more for depression than for anxiety disorders (×2 = 4.737, df = 1; p = .030).

### 3.3 The results on early-term side effects

All non-SSRI antidepressants were associated with psychological side effects and were found to be riskier in this respect than SSRIs. (×2 = 3.846, df = 1; p =.050, Cramer’s V = 0.20). Sexual side effects were more frequent in the group that had read the PIL (×2 = 5.678, df = 1; p = .017, Cramer’s V = 0.24), and been diagnosed with major depressive disorder (×2 = 8.653, df = 1; p = .003, Cramer’s V = 0.30) and the severity of these side effects were higher (U = 802.500, p = .003) in major depressive disorder. The distribution of the reported side effects and the relationship between the presence of side effects and other categorical variables are given in **Table 2**. Women reported more severe neurological, and autonomic side effects (**Table 3**). The total severity of side effects and psychological side effects were found to be higher in patients using other psychotropic agents in addition to antidepressants (**Table 3**). The relationships between the severity of side effects and other variables are presented in **Table 3**, and the correlations between the severity of side effects, age, symptom levels, and functionality are shown in **Table 4**.

**Table 2:** Comparisons of the reported frequency of side effect types between sex, diagnoses, and groups based on treatment characteristics.

Side effects (N = 100)	Psychological ×2; df n (%)	Neurological ×2; df n (%)	Autonomical ×2; df n (%)	Sexual ×2; df n (%)	Total ×2; df n (%)
Sex	1.263; 1	0.842; 1	1.836; 1	0.016; 1	1.327; 1
Women (n=72)	65 (90.3)	33 (45.8)	49 (68.1)	11 (15.3)	67 (93.1)
Men (n=28)	23 (82.1)	10 (35.7)	15 (53.6)	4 (14.3)	24 (85.7)
Diagnoses	0.140; 2	0.615; 2	1.796; 2	10.509; 2**	1.131; 2
ADs (n=38)	34 (89.5)	16 (42.1)	27 (71.1)	1 (2.6)	36 (94.7)
MDD (n=55)	48 (87.3)	23 (41.8)	32 (58.2)	14 (25.5)	49 (89.1)
Others (n=7)	6 (85.7)	4 (57.1)	5 (71.4)	0 (0.0)	6 (85.7)
Antidepressant Class	3.846; 1*	0.050; 1	0.932; 1	3.332; 1	2.790; 1
SSRI (n=78)	66 (84.6)	34 (43.6)	48 (61.5)	9 (11.5)	69 (88.5)
Others (n=22)	22 (100)	9 (40.9)	16 (72.7)	6 (27.3)	22 (100)
Treatment Status	0.072; 1	0.507; 1	2.157; 1	0.041; 1	0.683; 1
Beginning New Drug (n=78)	69 (88.5)	35 (44.9)	47 (60.3)	12 (15.4)	70 (89.7)
Changing The Drug (n=22)	19 (86.4)	8 (36.4)	17 (77.3)	3 (13.6)	21 (95.5)
Comorbid Physical Disease	1.007; 1	0.056; 1	0.041; 1	2.675; 1	0.221; 1
Positive (n=29)	27 (93.1)	13 (44.8)	19 (65.5)	7 (24.1)	27 (93.1)
Negative (n=71)	61 (85.9)	30 (42.3)	45 (63.4)	8 (11.3)	64 (90.1)
Number of Psychotropic Drugs	1.826; 1	2.077; 1	0.297; 1	2.939; 1	0.611; 1
Combination (n=34)	32 (94.1)	18 (52.9)	23 (67.6)	8 (23.5)	32 (94.1)
One Drug (n=66)	56 (84.8)	25 (37.9)	41 (62.1)	7 (10.6)	59 (89.4)
Additional Non-psychotropics	0.739; 1	0.031; 1	0.017; 1	1.513; 1	0.115; 1
Positive (n=27)	25 (92.6)	12 (44.4)	17 (63.0)	6 (22.2)	25 (92.6)
Negative (n=73)	63 (86.3)	31 (42.5)	47 (64.4)	9 (12.3)	66 (90.4)
Did the patient read the PIL?	0.041;1	0.009; 1	0.759; 1	5.678; 1*	0.123; 1
Yes (n=39)	34 (87.2)	17 (43.6)	27 (69.2)	10 (25.6)	35 (89.7)
No (n=61)	54 (88.5)	26 (42.6)	37 (60.7)	5 (8.2)	56 (91.8)
Did the patient need to reach his/her doctor?	0.395; 1	0.261; 1	0.003; 1	0.320; 1	0.000; 1
Yes (n=33)	30 (90.9)	13 (39.4)	21 (63.6)	4 (12.1)	30 (90.9)
No (n=67)	58 (86.6)	30 (44.8)	43 (64.2)	11 (16.4)	61 (91.0)
Medication Adherence	2.406; 1	0.673; 1	0.054; 1	0.038; 1	1.745; 1
Adherent (n=85)	73 (85.9)	38 (44.7)	54 (63.5)	13 (15.3)	76 (89.4)
Nonadherent (n=15)	15 (100)	5 (33.3)	10 (66.7)	2 (13.3)	15 (100)
(N = 65)	Psychological ×2; df n (%)	Neurological ×2; df n (%)	Autonomical ×2; df n (%)	Sexual ×2; df n (%)	Total ×2; df n (%)
Treatment Response	2.708; 1	3.055; 1	1.584; 1	0.058; 1	5.611; 1*
Responder (n=57)	53 (93.0)	24 (42.1)	41 (71.9)	9 (15.8)	55 (96.5)
Non-Responder (n=8)	6 (75.0)	6 (75.0)	4 (50.0)	1 (12.5)	6 (75.0)
Remission Status	1.643; 1	0.148; 1	2.185; 1	1.847; 1	1.059; 1
Yes (n=47)	44 (93.6)	21 (44.7)	35 (74.5)	9 (19.1)	45 (95.7)
No (n=18)	15 (83.3)	9 (50.0)	10 (55.6)	1 (5.6)	16 (88.9)

Ads, Anxiety Disorders; MDD, Major Depressive Disorder; SSRI, Selective Serotonin Reuptake Inhibitors.

*p<.05, **p<.01.

**Table 3:** Comparisons of the severity of side effects between sex, diagnoses, and the categories based on the treatment characteristics.

Side effects (N = 100)	Psychological mean; S. D. (t, p)	Neurological mean; S. D. (t, p)	Autonomical mean; S. D. (t, p)	Sexual mean; S. D. (t, p)	Total mean; S. D. (t, p)
Sex					
Women (n=72)	5.22; 3.693	1.08; 1.480	3.50; 3.950	0.29; 0.795	10.63; 7.507
Men (n=28)	4.89; 4.140 (0.387,.700)	0.54; 0.838 (2.324,.022*)	1.68; 2.374 (2.817,.006**)	0.25; 0.701 (0.243,.709)	7.93; 5.944 (1.703,.092)
Diagnoses					
ADs (n=38)	5.24; 3.989	0.89; 1.181	3.26; 3.569	0.03; 0.162	9.89; 6.375
MDD (n=55)	4.87; 3.454 (0.469,.640)	0.76; 1.036 (0.567,.572)	2.65; 3.627 (0.801,.425)	0.49; 0.979 (-3.452,.001**)	9.42; 6.941 (.0.336,.737)
Antidepressant class					
SSRI (n=78)	4.92; 3.907	0.96; 1.409	2.90; 3.436	0.23; 0.737	9.50; 7.382
Others (n=22)	5.86; 3.399 (-1.024,.308)	0.82; 1.140 (0.438,.662)	3.32; 4.444 (-0.474,.636)	0.45; 0.858 (-1.212,.228)	11.18; 6.389 (-0.970,.334)
Treatment Status					
Beginning New Drug (n=78)	4.99; 3.641	0.97; 1.329	2.96; 3.795	0.27; 0.733	9.71; 7.022
Changing The Drug (n=22)	5.64; 4.392 (-0.705,.483)	0.77; 1.445 (0.617,.539)	3.09; 3.221 (-0.146,.884)	0.32; 0.894 (-0.263,.793)	10.45; 7.854 (-0.431, 0.668)
Comorbid Physical Disease					
Positive (n=29)	5.07; 2.725	0.86; 1.060	3.14; 4.095	0.45;.910	9.93; 6.723
Negative (n=71)	5.15; 4.184 (-0.121,.904)	0.96; 1.458 (-0.320,.750)	2.93; 3.498 (0.257,.798)	0.21;.695 (1.261,.214)	9.85; 7.559 (0.058,.954)
Number of Psychotropic Drugs					
Combination (n=34)	6.35; 3.515	1.03; 1.381	3.68; 4.491	0.53; 1.107	12.03; 7.554
One Drug (n=66)	4.50; 3.820 (2.359,.020*)	0.88; 1.342 (0.526,.600)	2.64; 3.131 (1.351,.180)	0.15; 0.472 (1.903,.064)	8.76; 6.769 (2.201,.030*)
Non-psychotropics Use					
Positive (n=27)	5.07; 2.814	0.81; 1.001	2.41; 2.500	0.44; 0.934	9.11; 4.956
Negative (n=73)	5.15; 4.129 (-0.106,.916)	0.97; 1.462 (-0.517,.606)	3.21; 4.000 (-1.189,.238)	0.22; 0.692 (1.143,.260)	10.15; 7.854 (-0.785,.435)
Did the patient read the PIL?					
Yes (n=39)	5.85; 4.332	0.92; 1.326	3.82; 4.599	0.49; 0.997	11.67; 8.305
No (n=61)	4.67; 3.385 (1.435,.156)	0.93; 1.377 (-0.041,.968)	2.46; 2.826 (1.659,.103)	0.15; 0.543 (1.951,.056)	8.72; 6.157 (1.905,.061)
Did the patient need to reach his/her doctor?					
Yes (n=33)	5.36; 3.464	0.85; 1.460	3.48; 4.577	0.18; 0.584	10.45; 7.263
No (n=67)	5.01; 3.979 (0.429,.669)	0.97; 1.302 (-0.422,.674)	2.75; 3.125 (0.948,.345)	0.33; 0.842 (-0.898,.371)	9.58; 7.173 (0.570,.570)
Medication Adherence					
Adherent (n=85)	4.96; 3.853	1.00; 1.406	3.00; 3.726	0.31; 0.817	9.76; 7.495
Nonadherent (n=15)	6.07; 3.494 (-1.034,.303)	0.53; 0.915 (1.237,.219)	2.93; 3.390 (0.065,.949)	0.13; 0.352 (0.802,.424)	10.47; 5.181 (-0.348,.729)

S. D., Standard Deviation; ADs, Anxiety Disorders; MDD, Major Depressive Disorder; SSRI: Selective Serotonin Reuptake Inhibitors.

*p<.05, **p<.01.

**Table 4:** The correlations between the severity of side effects, age, symptom levels, and functionality.

Side effects	Psychological	Neurological	Autonomical	Sexual	Total
Age	-.281**	-.315**	.014	.036	-.177
Pre-treatment BDI score	.387**	.090	.148	.170	.298**
Pre-treatment BAI score	.252**	.119	.250*	-.071	.243*
Impairment Level of Pre-treatment Functionality	.201*	.101	-.025	-.147	.023

BDI, Beck Depression Inventory; BAI, Beck Anxiety Inventory.

*p<.05, **p<.01.

Linear regression analysis carried out in a single step revealed that pretreatment anxiety level was the significantly associated variable with the severity of early-term side effects. The results of the regression analysis are given in **Table 5**.

**Table 5:** Linear regression analysis of the factors associated with side effect severity.

Variables	Side effect severity ^a^			
	B	β	SE	T
Constant	10.646	–	4.634	2.297
Sex (Women vs. Men)	- 0.604	-.041	1.564	-0.386
Age	- 0.037	-.083	0.055	- 0.681
Diagnosis (Anxiety vs. Depression)	-0.092	-. 007	1.505	-0.061
Comorbid Physical Disease (None vs. Having Comorbidity)	- 0.821	-.057	1.826	- 0.449
Drug Class (non-SSRI vs. SSRI)	- 2.938	-.188	1.638	- 2.157
Number of Psychotropics (Only antidepressant vs. Combination)	2.707	.195	1.454	1.862
Pretreatment anxiety levels (BAI scores)	0.148	.283	0.054	2.482*

SSRI, Selective Serotonin Reuptake Inhibitor; BAI, Beck Anxiety Inventory; B, Unstandardized Coefficient; β, Standardized Coefficient; SE, Standard Error.

a R^2^ = .159, F (7, 92) = 2.298, p = .034.

*p <.05.

### 3.4 Medication nonadherence/drug discontinuation

Interviewers assessed adherence to antidepressant treatment by asking questions to understand whether there was an overlap between the psychiatrist’s recommendation and the patient’s use and whether the patient continued the treatment in phone calls and follow-up interviews with those who attended their scheduled evaluations.

Of the 100 patients screened for side effects, 33 (33.0%) reported a need to contact their doctor due to side effects, and 15 patients (15.0%) were nonadherent or discontinued their treatment before the control time.

Comparisons between nonadherent patients and those who adhered to their prescriptions showed no statistically significant differences in age, pretreatment anxiety levels, or the severity of side effects (respectively, U = 479.000, p = .126; U = 619.000, p = .858; U = 554.000, p = .420). However, nonadherent patients had significantly worse functionality scores before starting the antidepressants (U = 390.500, p = .013). The rates of nonadherence/discontinuation were similar across diagnostic categories, between patients who reported at least one side effect and those who did not, between men and women, between new antidepressant beginners and those switching medications, and across educational attainment groups (respectively, ×2 = 3.221, df = 1; p = .073; ×2 = 1.745, df = 1; p = .186; ×2 = 1.883, df = 1; p = .170; ×2 = 0.224, df = 1; p = .636; ×2 = 3.406, df = 2; p = .182). There was a higher number of nonadherent patients in the non-SSRI group, and the patients using a combination of psychotropic drugs (respectively, ×2 = 6.257, df = 1; p = .012, Cramer’s V = 0.25; ×2 = 5.316, df = 1; p = .021, Cramer’s V = 0.23). According to logistic regression analysis, the only statistically significant factor associated with nonadherence was the level of impairment in pretreatment functionality (**Table 6**).

**Table 6:** Logistic regression analysis of the factors associated with medication nonadherence.

Variables	Adherent vs. nonadherent ^a^		
	OR	95% CI	*p-value*
Constant	0.092		.022
Diagnosis (Anxiety Disorders vs. Depression)	0.271	0.058-1.254	.095
Drug Class (non-SSRI vs. SSRI)	2.259	0.582-8.779	.239
Number of Psychotropics (Only antidepressant vs. Combination)	0.361	0.103-1.273	.112
The Severity of the Side Effects	1.004	0.912-1.104	.939
The Impairment Level of Pretreatment Functionality	1.930	1.031-3.610	.040*

SSRI, Selective Serotonin Reuptake Inhibitor; BAI, Beck Anxiety Inventory; B, Unstandardized Coefficient; β, Standardized Coefficient; SE; Standard Error.

a χ2(5)=15.192, p =. 010.

*p <.05.

Of the 65 patients who attended follow-up, 41 (63.1%) had been able to continue their treatment regularly without describing any significant problems, 24 (36.9%) had additionally contacted their doctor due to intolerance and difficulty in adapting to treatment, and finally, the antidepressants of 14 patients (21.5%) who could not tolerate the drug were changed during the control appointment.

### 3.5 Treatment response

Among the 65 patients who completed follow-up, 7 of 35 patients diagnosed with depression did not respond to treatment (20.0%) and 27 (77.1%) achieved remission. Of 26 patients diagnosed with anxiety disorders, 1 (13.8%) did not respond to treatment, and 16 (61.5%) achieved remission. While the remission rate of the whole sample (both depression and anxiety disorders groups) was 72.3%, the rate of non-response to treatment was 12.3%. Then, we conducted the analyses examining associations between side effects and treatment response/remission as exploratory *post-hoc* analyses which were not pre-specified secondary outcomes. There was no difference in total side effect severity between responders and non-responders (t = - 1.221, df = 63, p = .226). However, the number of patients who reported at least one side effect was higher in responders (×^2^ = 5.611, df = 1, p = .018, Cramer’s V = 0.29). Patients achieving remission in one month reported higher severity of total side effects (U = 256.500, p = .015). When the side effects were categorized, remitters were found to only report higher severity of psychological side effects than non-remitters (U = 241.000, p = .007).

## 4 Discussion

This study focused on short-term side effects of antidepressants and medication adherence in a naturalistic setting. Our findings revealed a 21.5% nonadherence/discontinuation rate in one month, which aligns with previous research indicating that approximately 20% of patients discontinue antidepressants within the first month due to side effects or other factors (25). In addition, experiencing at least one adverse effect was very common (91%) in the first month of new antidepressant use. The pretreatment anxiety level was found to be a significant correlate of the reported severity of side effects. Although there is a complex interplay of various factors, our findings emphasized the importance of pretreatment functionality for medication adherence. Importantly, this study extends prior work by disentangling early nonadherence from baseline symptom severity and showing that baseline functional impairment, rather than symptom burden, was independently associated with early discontinuation in routine outpatient care. In addition, we identify unguided PIL reading as a potentially modifiable factor associated with early-onset sexual adverse effects. Finally, by demonstrating that early responders were more likely to report side effects, we challenge a purely “side effects as barriers” framing and suggest that some early adverse experiences may reflect early pharmacodynamic engagement rather than intolerance.

First of all, SSRIs were the most preferred antidepressant group as expected, in line with the recent recommendations (26). All patients in the non-SSRI group reported at least one side effect. Although there was previous research showing that emotional blunting, commonly associated with long-term SSRI use, may lead some patients to prefer non-serotonergic agents (5), when all psychological side effects were evaluated together, SSRIs still appeared to have the advantage of fewer short-term psychological side effects. In addition, non-adherence was found to be more common in non-SSRI users. The relatively safer psychological side effect profile of SSRIs may support their place as first-line whether changing or initiating the antidepressant regimen.

Psychological side effects were the most frequently reported adverse effect type for the whole group. In addition, we found a positive correlation between the severity of symptoms and psychological side effects. These results may point out the intertwining of psychiatric symptoms and side effects of antidepressants, leading to patients reporting these increased symptoms as side effects. Another finding that sexual side effects were reported more frequently in depression than other disorders may also be related to this condition. On the other hand, we found that sexual side effects were reported rarely, were negligible in the short term, and were not significantly related to treatment discontinuation. A previous study supportively revealed that sexual side effects were mostly reported at mild levels in the 6th week and were negligible in the 2nd week (27).

The analyses for determining the correlates of side effect severity point to polypharmacy as a related factor. Patients using multiple psychotropic agents at the same time reported greater total side effect severity, in line with the studies suggesting that overlapping pharmacodynamic pathways can intensify adverse events (28). Contrary to our expectations, no significant difference in side effect burden was observed between patients newly starting an antidepressant and those switching from another antidepressant, suggesting that changing antidepressants does not inherently worsen tolerability—unless other psychotropic drugs are involved. This result should be interpreted carefully, as the number of patients in the drug-changing group of our study was small. Moreover, the side effect profiles were found to be related to sex and age, although they were not associated with the total side effect severity. Women reported higher severity of neurological side effects, particularly headaches, in short-term use. This finding aligns with the previous study investigating antidepressant side effects in women with postpartum-onset major depressive disorder, which identified headache as the most common treatment-emergent adverse effect, despite a low overall incidence (29). Although there was limited data on sex-related differences in side effect profiles for short-term use, some studies reported that women had an increased risk for withdrawal symptoms, constipation, or weight gain, suggesting that women may be more susceptible to specific adverse effects in long-term use. In addition, we found correlations between younger age and the severity of psychological and neurological side effects. Previously, it was shown that younger people reported more side effects (30). One possible explanation is that younger patients may be more sensitive to side-effect patterns. For instance, Campos et al. (31) and Bet et al. (30), both with mean participant ages of 43 years, identified a higher prevalence of specific side effects, including dizziness and nausea, among younger individuals (30, 31). Another explanation for this finding is that lower doses may be recommended in the first month of treatment for older patients. The last possibility is that while older ages are often associated with a higher burden of side effects, such as anticholinergic side effects, and even cognitive impairment for older patients (32), these patients may not report their side effects.

Finally, our data revealed that pretreatment anxiety levels were associated with side effect severity, in line with reports suggesting that anxious distress can magnify adverse reactions and complicate clinical outcomes (33). One possible explanation is that heightened interoceptive awareness in anxious individuals amplifies the perception of bodily changes, intensifying or misinterpreting even mild side effects (34). These findings underscore the need to evaluate pretreatment anxiety symptoms, especially among patients with autonomic or somatic complaints, to optimize adherence and reduce premature discontinuation. Furthermore, anxiety-related physical symptoms, such as palpitations or gastric complaints, may overlap with side effects, making it hard for patients to distinguish between the two. Evidence suggests that most antidepressant side effects tend to lessen within the first few weeks of treatment, underscoring the importance of patient education and close monitoring during this critical period (35). By explaining the temporary nature of side effects and addressing patient concerns, clinicians may help improve adherence and reduce premature discontinuation. Another strategy may be the use of short-term additional drugs such as benzodiazepines to reduce anxiety and improve adherence, but the effectiveness of this strategy is still unclear. Although benzodiazepines are often stigmatized due to risks such as dependence, misuse, tolerance, and adverse effects including falls and cognitive impairment in older adults and therefore are generally not included as first-line treatment recommendations, they have demonstrated efficacy for short-term use in anxiety disorders (7). While they are not currently recommended as first-line monotherapy, short-term adjunctive use alongside antidepressants for anxiety disorders is still advised (7). However, the extent to which benzodiazepines are prescribed in real-world settings and how strongly their use is associated with treatment adherence remain insufficiently clear. In our sample, the use of benzodiazepines was additionally recommended for 15 patients at the initiation of antidepressant treatment. Of these patients, six were prescribed benzodiazepines with a planned dose reduction, five were advised to use them on an as-needed basis, and four were prescribed a fixed dose. Owing to the small number of benzodiazepine users in our sample, it was not possible to investigate these issues through further analyses. Examination of this topic in future studies appears warranted.

Antidepressant side effects can also be influenced by psychological and contextual factors, such as the nocebo effect, where negative expectations lead to adverse experiences independent of the drug’s pharmacological action (36). Learning paradigms, as demonstrated by Rheker et al. (13), further emphasize the role of conditioning and expectation in shaping patients’ experiences of side effects (13). In this context, our study adds novel, clinically actionable information by suggesting that reading of patient information leaflets (PILs) may be associated with the early onset of sexual side effects: an association that, to our knowledge, has not been systematically tested in prior naturalistic outpatient studies.

This relationship may be two-tailed. This finding may be related to the increase in performance anxiety in those who read the PIL or the fact that those who experience these side effects may have explored the side effects in the PIL However, contrary to our expectations, this attitude did not appear to be a factor that increased the general severity of side effects. Educating patients about the nocebo effect has been shown to lessen the perceived burden of side effects and foster a deeper understanding of positive treatment expectations (37). Such approaches, integrated into psychoeducational interventions, may help mitigate the disruptive impact of PIL reading and ensure patients remain engaged throughout their treatment.

Another aim of the study was to explore the relationship between the side effects of antidepressants and medication adherence. Previous studies have highlighted the importance of adherence in enhancing treatment response, suggesting that adherence plays a more critical role than dosage adequacy (38). In previous studies, side effects such as somnolence, dizziness, and headaches were shown to increase dropout rates of SSRI treatment (35, 39). Psychological side effects, such as emotional blunting, were also known to influence antidepressant adherence (5) Nevertheless, we did not find a significant relationship between the overall severity of side effects and medication adherence. However, our study underscores the relationship between pretreatment functional impairment and medication adherence. The inability to perform routine tasks in work, family, and social environments may affect essential functions such as medication adherence, leading to treatment discontinuation. This finding supports non-adherence patterns observed in other chronic diseases (40).

Moreover, beyond the patient-related factors, our findings also highlight the possible role of physician-patient communication in mitigating premature dropouts. In our sample, all nonadherent patients reported the need to contact their psychiatrists for guidance. This pattern underscores the possible role of timely, accessible physician-patient communication in preventing complete dropout and supporting re-engagement with treatment. Our findings are consistent with earlier studies indicating that effective communication and psychoeducational tools can improve adherence and reduce discontinuation rates (35, 41). In line with these studies, there were recommendations on providing a short-term treatment plan and scheduling a follow-up within 2–4 weeks to assess tolerability, adherence, and early signs of response (42). From a service-delivery perspective, our results highlight the first month as a high-risk window in routine outpatient care and support the rationale for testing targeted early-contact strategies, especially for patients with lower baseline functioning and higher anxiety.

Another finding of this study was that a higher number of responders to antidepressants reported at least one side effect. In addition, early-term remitters reported higher severity of psychological side effects. Consistent with our findings, some previous studies reported that patients who ultimately respond to antidepressants experienced earlier side effects, suggesting that mild to moderate adverse events may reflect underlying pharmacodynamic processes crucial for therapeutic efficacy (43). However, there were controversial findings from different studies. Fabbri et al. (44) observed fewer side effects in early responders, while Braund et al. (45) emphasized that the perceived burden—rather than the frequency—of side effects more strongly predicted negative (44, 45). Evidence from novel therapies, such as psilocybin or ketamine, suggests that mild, short-lived side effects can signify engagement of key pharmacodynamic pathways (46). These findings may highlight the need to determine an optimum level of side effects and distinguish side effects that indicate therapeutic activity from those associated with intolerance and interfere with adherence. Accordingly, viewing side effects as potential indicators of treatment activity, rather than simply obstacles, and informing patients may contribute to optimizing adherence. Studies with larger samples may be beneficial for understanding the effects of different side effect profiles on treatment response.

Although the study’s prospective design and naturalistic approach offer valuable insights into the short-term effectiveness and tolerability of antidepressants, it has some limitations. First, we did not explore the dosing effects by dose equivalence analysis, assuming the minimum effective doses were given when initiating treatment. In addition, details regarding the speed of dose titration, the timing of administration, and whether medications were taken with or without food were not assessed, nor were their associations with side effects or nonadherence analyzed. Second, the severity of symptoms relied on self-report scales, which may be subject to recall or reporting biases. Third, the sample size was relatively small for such a heterogeneous group, precluding in-depth subgroup analyses—such as examining the relationship of nonadherence with individual side effects or comparing each antidepressant separately. Although focusing on the first month allowed us to capture short-term effects, this timeframe may be too early to determine definitive treatment response, limiting the generalizability of our findings to long-term outcomes. Additionally, we did not objectively verify medication adherence (e.g., pill counts, electronic monitoring), relying instead on patient self-reporting. Further, the rate of follow-up losses was high, partly due to the February 6th, 2023, Kahraman Maras–Turkey earthquake, and we lack the reasons for most dropouts. Those who dropped out were older and reported lower baseline symptom severity, but we could not assess how these differences might have influenced our results. In addition, multiple exploratory comparisons were conducted, which may have increased the risk of type I error. Further, this study was conducted in a single-center setting, which may limit the generalizability of the findings to other clinical or health care settings with different patient populations or treatment practices. Finally, although we did not observe a difference in nonadherence rates between patients who had previously used and subsequently switched antidepressant treatment and those who were using antidepressants for the first time, prior exposure to similar treatments might still be another factor that may influence attitudes toward adherence to antidepressant treatment. Therefore, analyzing both groups together should also be considered a limitation of this study. In conclusion, there are a significant number of patients who cannot continue antidepressants in the early term. Informing patients with high pretreatment anxiety levels about side effects and reducing anxiety before starting antidepressants may be beneficial for side effect management. In addition, incorporating routine functionality assessments and informing patients about side effects can support personalized treatment strategies and reduce early discontinuation and delayed recovery. Future research should validate these findings with larger, more diverse samples and explore targeted interventions—such as tailored patient education and shared decision-making—that specifically address impairments in functionality and level of anxiety. These efforts will further optimize antidepressant therapy and adherence in diverse healthcare settings.

## Data Availability

The raw data supporting the conclusions of this article will be made available by the authors, without undue reservation.
